# In good times and in bad: How plasma cells resolve stress for a life-long union with the bone marrow

**DOI:** 10.3389/fimmu.2023.1112922

**Published:** 2023-03-08

**Authors:** Carolin Ulbricht, Yu Cao, Raluca A. Niesner, Anja E. Hauser

**Affiliations:** ^1^ Department of Rheumatology and Clinical Immunology, Charité - Universitätsmedizin Berlin, corporate member of Freie Universität Berlin and Humboldt-Universität zu Berlin, Berlin, Germany; ^2^ Immune Dynamics, Deutsches Rheuma-Forschungszentrum (DRFZ), A Leibniz Institute, Berlin, Germany; ^3^ Biophysical Analysis, Deutsches Rheuma-Forschungszentrum (DRFZ), A Leibniz Institute, Berlin, Germany; ^4^ Dynamic and Functional in vivo Imaging, Veterinary Medicine, Freie Universität Berlin, Berlin, Germany

**Keywords:** metabolism, bone marrow, autophagy, long-lived plasma cells (LLPC), calcium, antibody production

## What makes a plasma cell long-lived?

The plasma cell (PC) is the terminal differentiation state of B lymphocytes, phenotypically characterized by an enlarged granular cell body with a highly productive endoplasmic reticulum (ER) and strict energetic requirements to maintain their main function: antibody production. Despite being mitotically quiescent, PC are able to keep up high anabolic activity for months, up to several years. Early studies on “immunoglobulin producing cells” not yet characterized in detail at this point in time suggested an antibody production rate of several ten million molecules per hour and cell (1, 2). When resting B cells are activated in or outside of germinal centers (GC) giving rise to plasma blasts (PB), they rapidly undergo metabolic reprogramming and increase glycolysis and oxidative phosphorylation (OXPHOS) rates (3). From there, they can develop into either short-lived PC (SLPC) or home to the bone marrow (BM), where they become resident (4, 5), long-lived PC (LLPC) (6, 7). However, whilst some cellular and molecular components of the PC niche have been identified (8, 9), the factors maintaining those cells productive at high levels are less well known. Even telling apart SLPC from LLPC is not an easy job. Microanatomical location seems not to be a definite criterion, since affinity-matured PC and LLPC were also found to persist in spleen (10, 11) and in inflamed tissue, in mice and humans alike (12, 13). While one study in mice claimed that LLPCs are produced late during GC responses (14), results from a recent publication indicate that murine LLPC are also formed in early phases of the GC response (15), with some LLPC having undergone no affinity maturation at all (16). Furthermore, evolutionary conserved surface markers for PC, let alone LLPC, are missing. The majority of PC do express B cell maturation antigen (BCMA) and CD138 in mice and humans (17–20), however, there is also substantial CD138 heterogeneity found in human cultured BM aspirates (21). There is further no unique distinctiveness of surface markers for LLPC, which is also true for transcriptional programs (22). Most prominent, the expression of *Prdm-1, Mcl1, Irf4 or Xbp1* are common requirements (15, 23). Additionally, microRNA miR-148a was identified in murine LLPC serving metabolic regulation (24). Very recently, after pre-sorting of murine LLPC from SLPC according to scRNA-sequencing-assisted clustering of PC subsets (23), the adhesion molecule EpCAM and a lack of the chemokine receptor CXCR3 were identified as characteristics of IgG LLPC in mice. This approach also illustrates that considerable effort is to be undertaken in order to point out these very rare cells from a heterogeneous parent population.

Investigating metabolic features as an additional deterministic layer of plasma cell development could help to simplify the identification of LLPC. Since the metabolism of a B cell becoming a PC needs to change quite dramatically, we suggest taking the activation of metabolic enzymes, glycolysis and respiration rates and the import of nutrients into account. Metabolic pathway properties at different stages of the maturation and differentiation process may be of an – in part epigenetic – advantage for cell longevity. For example, murine studies in pre-B cells have linked receptor signaling with the regulation of chromatin accesibility (25). In Th17 cells, the accumulation of the citric acid cycle metabolites 2-hydroxyglutarate and acetyl-CoA confers transcriptional regulation via histone modification (26, 27). Whether such cues are able to lastingly imprint the prerequisites of a long-lived phenotype during B cell activation, and if so, for how long, is not known. The fact that serum half-lives of antibodies induced by different viral antigens (Ag) are heterogeneous [e.g., 200 years for measles, 50 years for varicella zoster, 11-19 years for diphtheria and tetanus (28)] is suggesting a dependence of PC longevity from initial triggers (29). Viewed from the B cell side, high-affinity BCRs seem to generate a selection advantage through increasing OXPHOS (30). Similarly, differential CD19 expression could link initial signaling through Ag activation and metabolic rewiring. CD19, on the one hand, is enhancing BCR induced calcium flux to overcome a proposed Ag threshold (31). On the other hand, CD19 in mice is negatively regulating the activity of phospho-inositide-3-kinase (PI3K), an enzyme crucial for initializing metabolic pathways supporting proliferation and survival. In B cells, CD19 deficiency leads to a 50% increase in PI3K activity compared to CD19 sufficient counterparts (32). Since PI3K activity is also a pro-survival factor dependent on stromal cell contact and important for the regulation of mitochondrial integrity and ER-stress in LLPC (33), CD19^-^ PC might have an advantage in coping with metabolic stress. In support of that, the downregulation of CD19 was identified as a hallmark of longevity in human plasma cells (34, 35).

## A closer look on PC metabolism

**Figure 1:**
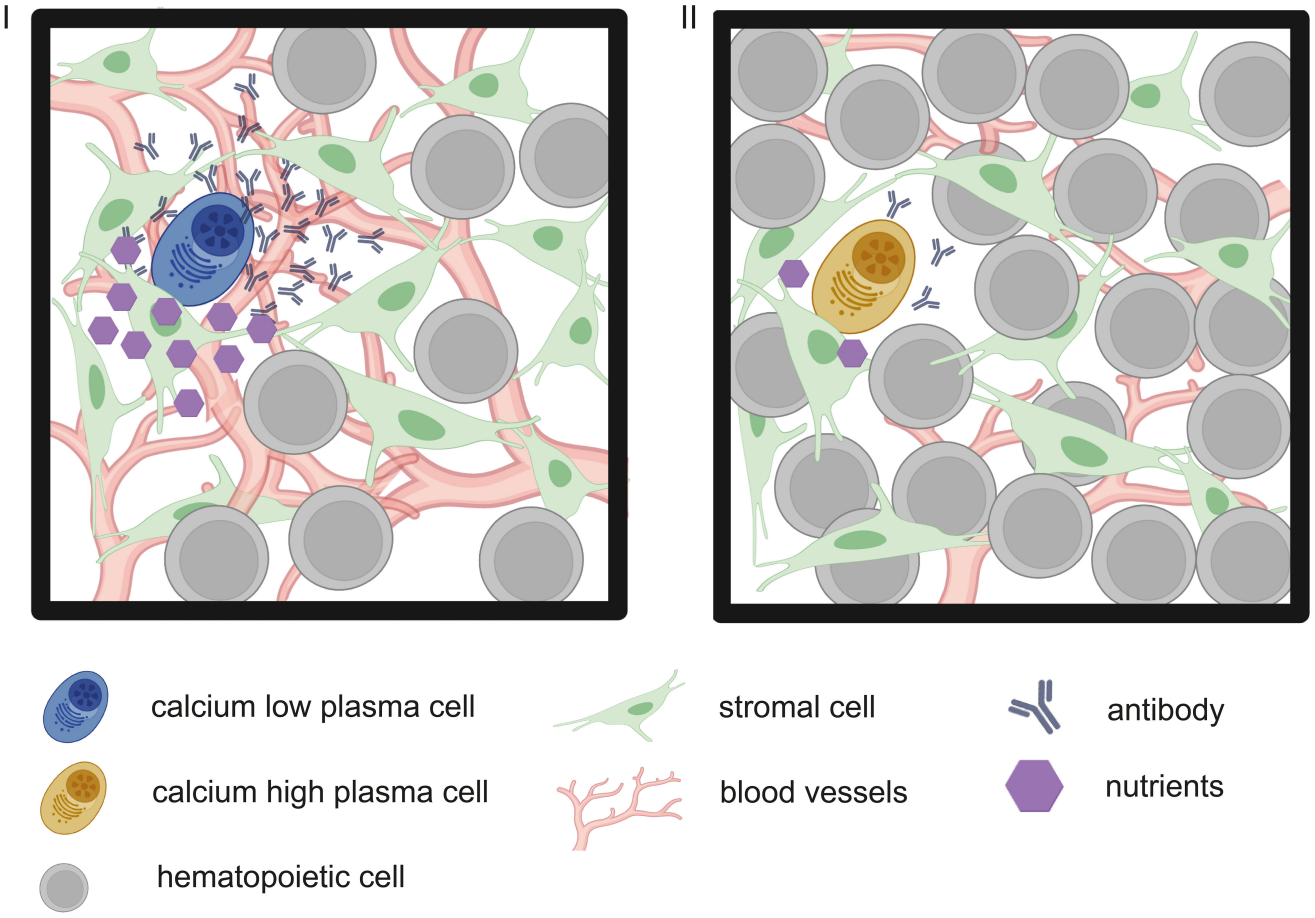
Graphic representation of two hypothetic metabolic states of PC within the BM, depending on niche composition. In this model, PC within their stromal cell niche (green mesenchymal stem cells) are low in cytoplasmic calcium (I, blue PC) in times of sufficient nutrient supply (purple hexagons), e.g. due to a dense vessel network (red branches) and only few other hematopoietic cells (grey), competing for nutrients. Calcium-low cells produce antibodies at high rates. Vice versa, in times of metabolic stress (II) through decreased nutrient supply due to hematopoietic nutrient sinks or vessel remodeling (5), antibody output is decreased.

BMPC mostly depend on OXPHOS, using amino acids, especially glutamine, for their carbon demand; despite significant upregulation of the glucose transporter GLUT1 (3). The latter is explained by the fact that glucose in PC is primarily used for antibody glycosylation. However, in times of decreased nutrient availability, PC can also metabolize the imported glucose into pyruvate driving mitochondrial respiration (36). Missing glucose, however, would slow or shut down the hexosamine biosynthesis pathway producing N-Acetyl-glucosamine (GlcNAc), as shown in cancer cells (37, 38). Since it is an essential structural component of protein glycosylation patterns, missing GlcNAc would mean failure in protein modification and trafficking (39). This mechanism has so far been only proven for tumor-associated proteins, but supposedly would have severe functional consequences for antibodies, too (40). Antibody misfolding, hindered degradation of misfolded antibodies or insufficient export from the ER induces ER stress that goes along with calcium efflux, affecting phosphorylation of metabolic gate-keepers like adenosine monophosphate (AMP)-activated protein kinase (AMPK) and mechanistic target of rapamycin (mTOR) (41–43). In fact, LLPC do import larger amounts of the fluorescent glucose analog 2-Deoxy-2-[(7-nitro-2,1,3-benzoxadiazol-4-yl)-amino]-D-glucose in mice, albeit via a still unidentified transport system (44, 45). If glucose does becomes limiting and falsely glycosylated antibodies are produced, then the cells are equipped with a rescue mechanism called the unfolded protein response (UPR). Ultimately, UPR is leading to autophagy that mobilizes additional energy sources by recycling of cellular building blocks from organelles like mitochondria and ER, a key process found indispensable for PC survival (46). The importance of UPR and autophagy in PC is further illustrated by the fact that PC signature genes such as *Xbp1, Ire1* and *Atg6* are devoted to these processes, though the latter is silenced in terminally differentiated PC (47, 48). Of note, the term UPR is misleading, as this process is primarily a timed orchestration of developmental factors preparing and supporting the B cell- to plasma cell-transition rather than an emergency response to unfolded proteins (49). Still, high-rate antibody output is considerably challenging the cellular machineries of posttranslational modification, folding and trafficking that has to withstand times of crises. Thus, we question a constant secretion rate and instead propose the dynamic regulation of energy metabolism and antibody output as a feature essential for PC longevity (**Figure 1**).

## Intracellular calcium as integrator of stressors

Calcium-mediated BCR signal strength has been known to encode different B cell fates by regulating downstream transcription and mTOR activity for quite some time (50–52). Unexpectedly, employing functional intravital imaging in mice, our group was able to detect also Ag-specific PB displaying vivid calcium fluctuations within a comparatively high concentration range (>500 nM) among otherwise calcium-low (<200 nM) peers (53). Ruling out the possibility of residual BCR signaling or cytokine receptor signaling, we sought for an alternative explanation for the existence of a calcium-high population among PB. Possibly, metabolic stress signals as a consequence of competition for nutrients in the densely packed medullary cords lead to transient calcium increase in the cytoplasm, likely through mitochondrial release (54, 55). Second, high production rates of antibodies induce ER-calcium release alongside ER-stress, initiating calcium replenishment from the extracellular environment. One proposed downstream effector of increased cytoplasmic calcium is AMPK. In times of energy crises, B lineage cells rely on AMPK for survival and functional maintenance (56, 57). AMPK is binding energy-poor AMP when its abundance is high – meaning adenosine triphosphate (ATP) amounts are low. AMP binding leads to AMPK phosphorylation and therefore activation. Studies with fully nutrient-supplied T cells, however, have shown that AMPK phosphorylation can also occur allosterically via calcium-calmodulin/dependent kinase kinase β (CamKKβ) (58, 59). Leakage of calcium ions from mitochondria and/or ER could possibly contribute to reaching a calcium concentration threshold for this pathway to take hold. Importantly, chronic calcium exposure has been found to inhibit AMPK in muscle cells via increased phosphatase activity, in a setting where the cells were additionally treated with 5-aminoimidazole-4-carboxamide ribonucleotide (AICAR), an AMP analog (60, 61). Calcium oscillations, however, activated AMPK. One of the main functions of active AMPK is to inhibit mTOR activity *via* phosphorylation of adapter molecules (62). Since mTOR inhibition leads to autophagy, stress-induced calcium release into the cytosol would directly lead to switching on mechanisms resolving stress or at least bridging the time until more favorable environmental conditions arise.

But why is it that PC would need such mechanisms in the first place? Given the BM is an organ with high cellular turnover due to hematopoiesis and a tissue undergoing profound age-related changes, it is evident that a certain degree of heterogeneity in the survival niches exists in space and time.

## The PC niche and PC turnover models

The BM PC niche fulfills two basic requirements: promoting PC survival and serving as a foundation for adhesion. The stromal cell scaffold of the BM, together with several types of accessory cells, provides the main pro-survival cytokines A proliferation inducing ligand (APRIL), IL6 and CXCL12, the latter being also the chemoattractant guiding PB from secondary lymphoid organs to the BM (63, 64). CXCL12 is sensed by the PC *via* CXCR4, and tight PC adhesion within the niche is accomplished by VLA4 and LFA1 pairing with VCAM1 and ICAM1 of stromal cells, respectively (65, 66), as well as CD28 with CD80/86 (67, 68). CD28 expression supports LLPC survival by maintaining metabolic fitness *via* reactive oxygen species (ROS)-dependent signaling and IRF4 activity (69). APRIL directly supports PC survival and can be sensed by the PC *via* TACI and BCMA (70–72), an interaction also critical for differentiation of B cells into PC and therapeutic target in autoimmunity and multiple myeloma (MM) (73–75). Interestingly, homing can take place also in APRIL-deficient mice (65), so the pro-survival effect is likely independent from direct cell contact. Further, CD138, next to functioning as an adhesion molecule and receptor, can augment the scavenging of APRIL and IL-6 *via* binding to its heparan sulfate-chains on the cell surface and therefore support survival (76–80).

The general perception of the niche is that it consists of static (the stroma) and dynamic components, i.e. accessory cells providing soluble cytokines, such as granulocytes, megakaryocytes and others (9). Further, the number of niches is finite. PC will home to their niche persistently, unless driven away by inflammation and being outcompeted by newly formed PC (81). Models of PC turnover discussed include the random turnover model, in which newly formed PC replace older ones by chance, and the deterministic model, in which PC originating from certain B cell clones have a greater intrinsic (or imprinted) capability of homing and replacing than others. The problem of both these models is that they assume niches are changing neither in quality nor in number. However, a growing number of studies are reasonably challenging these notions, as discussed below.

## Heterogeneity in the BM

The reason that PC are considered static in their survival niche is probably the time scale on which we observe them in *in vivo* imaging studies. Using time-lapse imaging of up to 12 hours, Benet et al. could show that PC indeed experience phases of increased displacement between marrow regions, interrupted by periods of sessility (82). In addition, they observed clusters of PC in certain areas. While on the one hand this fact disproves the paradigm that PCs always remain alone in the same niche, it also means that the extrinsic conditions in the BM are divided into zones; those that promote movement and those that retain PCs (63). These conditions can also change, presumably on the level of cytokine abundance and/or receptor expression.

For the hematopoietic stem cell (HSC) niche, a heterogeneous architecture has been known for a long time (83). It has been discussed that HSC and leukocytes share in part similar features of their respective interaction with stromal cells (84). Strikingly, they often co-localize with PC and require the same cytokine, CXCL12 (85). Therefore, we would like to argue that the following observations made for HSCs may also apply to the LLPC BM niche, however, this still awaits confirmation. Quiescent HSC reside in endosteal regions, whereas sinusoidal areas are characterized by dynamic turnover and leukocyte trafficking. Local oxygen concentration gradients might in part cause this heterogeneity, as demonstrated by direct oxygen concentration measurements using phosphorescence lifetime imaging (86). These researchers stressed that the BM as a whole is a hypoxic organ despite being highly vascularized, however they found a steep oxygen drop from the endost to perisinosoidal regions, and with growing distance to blood vessels. Notably, areas densely packed with cells appear to be downright oxygen sinks, “reminiscent of solid tumors” (86). Further, variations in the permeability of blood vessels were identified to cause these local oxygenation differences and ROS load of the cells, as indicated by indirect determination with pimonidazole staining and Hif1-a expression in HSC (87). Since the blood flow transports all kinds of nutrients into the highly metabolically active BM, the same might be true for carbon sources needed for PC homeostasis. Further, interstitial calcium concentrations are low in regions with new bone material deposition and hematopoietic progenitors are absent in these regions, leaving the question open to what extent extracellular calcium concentration influences intracellular calcium levels, and therefore cell turnover *in vivo* (88). These findings illustrate how a microanatomical and metabolite heterogeneity consequently leads to a differential distribution of BM cells. Assuming the same is true for the plasma cell niches is only reasonable. However, up till now, no adequate tools for spatially and temporally resolved metabolic analyses within the BM are available *in vivo* (8).

## Heterogeneity as a result of BM dynamics

One approach to be expanded towards functional metabolic analysis is longitudinal intravital imaging of the BM (LIMB) (5, 89). Using this novel micro-endoscopic technique, our group was able to show that during homeostasis, vessel distribution, volumes and numbers are dynamically changing over time in the femoral marrow. Mice carrying the LIMB endoscope were repeatedly imaged for up to 6 weeks, revealing constant vessel remodeling. This temporal heterogeneity can be explained by several mechanisms: First, life-long exposure to gravity and exercise contribute to bone remodeling and immune cell health, as highlighted by studies under microgravity (90, 91). This effect might very well influence niche dynamics over time. Further, injuries might compromise the integrity of niches, leading to revascularization and cell population redistribution (89). Particularly, mechanosensing in macrophages mediated by the Piezo family of ion channels plays a pivotal role in bone regeneration following irradiation, and mechanosensing also impacts on the HSC niche and number of common lymphoid progenitors (92–94).

Furthermore, the alternation of day and night contributes to temporal heterogeneity of BM microenvironments through circadian gene and protein expression. This very likely affects plasma cell niches, too. The cellularity of the BM in mice and men (although they experience anticyclic rhythms since mice are nocturnal and humans usually not), has been shown to change quite drastically during any 24h. While structural bone remodeling usually peaks 1 hour after daybreak (95), blood replenishment and immune cell circulation even peak two times a day, regulated by two bursts of norepinephrine and tumor necrosis factor (TNF) (96). HSC cell trafficking to the blood was shown to be dependent on rhythmic CXCL12 oscillations from BM stroma, orchestrated by signals from the central nervous system (97). Furthermore, the homeostatic clearance of neutrophils within the marrow provides cues that directly act on the hematopoietic niche (98). That these mechanisms also have functional implications on the adaptive immune response has been demonstrated by the induction of experimental autoimmune encephalomyelitis, a mouse model for multiple sclerosis, at different time points per day (99). When cell counts were high in the periphery (end of day), mice showed dramatically faster disease progression than at the end of the night. It has been argued that this is in part because immune cells are reentering the marrow at night. Such daily turnover of cells in the microenvironment of PC niches is likely contributing to pressure-induced and nutrient supply-induced stress signals, requiring a certain amount of metabolic flexibility. However, longitudinal studies on BM resident cells have yet to be performed. Interestingly, Golan et al. hypothesize that quiescent HSC remaining in the marrow need to “train” their metabolism daily through circadian rhythms, in order to be ready for immediate activation during alarm situations (96). Of note, apart from gene expression and posttranslational modification, phosphorylation is another layer of metabolic regulation readily accessible and rapidly controllable by circadian signal transduction (100). Since many kinases are also calcium dependent enzymes, we expect to see periodic fluctuations in BMPC cytoplasmic calcium as well.

Along with changing daylight also goes diurnal food intake, which influences time-dependent availability of nutrients to the BM. A study investigating the effect of fasting on monocytes found a reduced inflammatory activity as an effect of AMPK-mediated sensing of low energy equivalents, and hence reduced BM egress (101). In the gut, fasting leads to a reduction in B cell numbers in Peyer’s patches, as well as egress of naïve B cells, which, in accordance with the response during the night (or during day in nocturnal animals), retreat to the BM for the time of nutrient deprivation (102). After refeeding, the cell pool is altered. The intracellular consequence of fasting-refeeding, i.e. the transition from low to high glucose levels, is remodeling mitochondria-ER contact sites and calcium ion exchange between the organelles (103). As seen from the circadian oscillatory IgA response in the gut, feeding times also directly impact on PC (104).

To stress the time component of BM heterogeneity, it should also be mentioned, that ageing disrupts bone marrow composition and circadian regulation, as shown for macrophages (105). The phenotypic appearance of BM in old age is characterized by a high proportion of adipocytes, which may well have a significant influence on the number and availability of niches. In fact, the impairment of hematopoiesis by fat deposits has been shown (106). Under this regard, it should also be discussed how results from mouse models can be applied to humans, since the murine BM commonly studied is juvenile and its human counterpart only exists in infancy.

## Impact of niche dynamics on humoral immunity

Taken together, BM microenvironments are most likely subject to heterogeneity in space and, perhaps even more important for cell survival, in time. How will this affect plasma cell longevity? LLPC need to be equipped with mechanisms coping with stress signals in a flexible manner. In other words, LLPC will have to translate extracellular stimuli into intracellular responses, eventually affecting metabolism, to a point that ensures reduced energy consumption, but only to an extent not leading to cell death. Stressing this point, the sensitivity of GC B cells and PB to mTOR inhibition by rapamycin stands in contrast to the resistance of LLPC to the drug (107). Instead of dying, LLPC reduce antibody production, as shown in rapamycin-treated mice, ameliorating autoimmunity. After discontinuation of rapamycin injections, antibody levels reinstall to values before treatment, highlighting the reversible capacity of antibody secretion in LLPC. One feature of metabolic flexibility could be for instance a greater spare respiratory capacity seen in LLPC vs SLPC, meaning the difference from basal respiration to the ability to increase mitochondrial electron throughput under stress (36). The manipulation of stress-resolving mechanisms could also have implications for the development of new therapies in the treatment of malignant PC-mediated diseases, like MM or auto-antibody mediated inflammatory diseases (108). These diseases have in common that their causative agents, antibody-producing cells, cannot be eliminated by classical B cell-depleting therapies or immunosuppressive agents (109). The recognition that protein-secreting cells are highly dependent on an intact ER led to the use of the proteasome inhibitor bortezomib in MM and in several autoimmune diseases (110–112). For MM therapy, targeting glutamine metabolism has proven promising (113, 114). However, since PC have developed the aforementioned sophisticated exit strategies to conquer metabolic stress, for complete success of these therapies it will be necessary to identify and target key molecules in the stress-response pathways, probably in combination. For example, *in vitro* and in mouse models, a synergistic anti-tumor effect of mTOR inhibition with rapamycin and bortezomib has been reported (115).

Vice versa, vaccine development would profit massively from knowing how to trigger longevity in PC and thus ensure lasting protection. Despite a continuously growing global vaccine market that by now has crossed the 100 billion dollar threshold and is expected to gain another 60 billion over the next four years (116), efforts to decipher the true mechanistic nature of durable antibody responses remain low. Advances come from the investigation of immunity induced in response to virus-like particles (117, 118). Schiller et al. argue that presumably the molecular form of Ag decides over induction of LLPC, also discussing an Ag-imprinting mechanism (119, 120). They found that vaccines against human papilloma virus (HPV), gain their remarkable durability from specialized structural features of the protein sequence, naming especially repeats with uniform spacing capable of activating several BCRs of both IgM and IgD subtype, thus inducing an exceptionally strong signal transmitted by IgD (121). Recently, TLR7 signaling has been shown to support the establishment of a favorable vaccination outcome (122). Some time ago, also TLR9 was identified in playing a role in B cell activation leading to stabilization of glycolytic activity, and the circumvention of mitochondrial depolarization by increased intracellular calcium (123, 124). Further, vaccination failure due to a lack of Ag-specific LLPC and an accumulation of SLPC in the BM has been linked to a gain-of-function mutation in *Cxcr4* common in warts, hypogammaglobulinemia, infections, and myelokathexis (WHIM) syndrome and Waldenström’s macroglobulinemia (125). This could be explained with enhanced mTOR signaling promoting extrafollicular PC differentiation and BM homing, as seen in a T-independent setting (126). These results are once more stressing a close connection between the sensing of environmental factors by innate immune receptors, BCR- and cytokine signaling and the homeostasis of controlled metabolic properties in establishing LLPC.

## Conclusion

In summary, understanding the principles driving differentiation of PC into the either short-lived or long-lived phenotype will be crucial for future therapy as well as vaccine development. Additionally, the factors that maintain LLPC in BM or inflamed tissue survival niches and their variability need to be taken into account (**Table 1**). Unfortunately, unambiguous identification of LLPC is challenging, requiring multiple analyses of surface markers and transcription factors. Identification by lifetime might be possible by time-stamped fate-mapping (15, 127). To also study the function of LLPC in their natural environment, new longitudinal imaging techniques are urgently needed. One approach to pursue is the monitoring of metabolic parameters and their change over time or in response to stimuli that mimic metabolic stress situations, for example with a combination of LIMB and fluorescence lifetime imaging and enzyme mapping (5, 53, 128, 129). Further, because the BM environment is constantly changing due to time of day, pressure changes in the tissue, aging or diseases and along with it the cellular composition, the niches themselves will likely also change. Thus, LLPC will encounter different environmental challenges, namely supply with nutrients, oxygen or cytokines, among others. Interestingly, though we still need more research on what triggers longevity in LLPC, existing data is pointing toward an imprinting mechanism by which some PC are favoured over others to become LLPC. The imprinted features are most likely metabolic regulators for the resolution of stress. Regulation could for instance be achieved dynamically by periodically cycling processes of autophagy and UPR, in connection with changing intracellular calcium levels and fluctuations in antibody output.

**Table 1:** Extrinsic and intrinsic factors impacting on survival and longevity of BMPC (alphabetical order).

receptor interaction	ref.	metabolic features	ref.	turnover	ref.	potential Imprinting	ref.
CD138-heparan sulfate	(76–80)	AMPK-mTOR axis	(40–42, 55, 56)	age	(104, 105)	activating BCR signal strength	(49–52)
CD19-PI3K	(31–35)	autophagy	(46)	circadian rhythm	(95–98)	timing of immune reaction	(99)
CD28-CD80/86	(67, 68)	epigenetic regulation	(25–27)	gravity	(90, 91)	TLR signaling	(122–124)
CDCR4-CXCL12	(63, 64)	cytoplasmic calcium	*to prove*	inflammation	(81)	type and structure of antigen	(28, 29, 120, 121)
LFA1-ICAM1	(65)	OXPHOS	(3, 30)	injury	(89)		
TACI/BCMA-APRIL	(68–75)	oxygen partial pressure	(86, 87)	interstitial calcium	(88)		
VLA4-VCAM1	(66)	proteasome activity	(110–112)				
		ROS	(69)				
		sugar import	(36, 44)				
		UPR	(47–49)				

## Author contributions

CU, YC, AH, and RN conceptualized the storyline. CU drafted the manuscript. AH, RN, and YC proof-read and complemented the manuscript. All authors contributed to the article and approved the submitted version.
